# Admission to a psychiatric unit and changes in tobacco smoking

**DOI:** 10.1186/1745-0179-4-12

**Published:** 2008-05-06

**Authors:** Suzy Ker, David Owens

**Affiliations:** 1 Briary Unit, Harrogate District Hospital, Harrogate, North Yorkshire, UK; 2 Academic Unit of Psychiatry and Behavioural Sciences, University of Leeds, Leeds, UK

## Abstract

Smoking and withdrawal from smoking complicates the assessment and treatment of mental illness. We aimed to establish whether psychiatric inpatients smoke different amounts after admission than beforehand and, if so, to find out why. Forty-three inpatients on a working age adult psychiatry ward completed self-report questionnaires about smoking habits. Those who smoked a different amount after admission had a follow-up interview to find out why they thought this had occurred. The interview incorporated qualitative and quantitative aspects which were analysed accordingly.

Fifty-six percent of participants were smokers before admission, rising to 70% afterwards. Of the smokers, 17% smoked less after admission, and 63% smoked more. The average number of cigarettes smoked per person per day increased from five to thirteen. The main reasons for smoking more were boredom, stress and the wish to socialise.

## Findings

Over the past 40 years, there has been a general decline in smoking [1] yet there has been little change in certain subgroups, among them people with mental illness [2,3]. Smokers who have mental illness require higher levels of antipsychotic medication [4,5], report more fatigue [6] and are more likely to have a history of suicide attempts [7,8]. Effects of smoking and withdrawal can complicate assessment and treatment of mental illness and make diagnosis more difficult.

We became increasingly aware of heavy smoking in psychiatric units, representing a public health problem and a worry about the future care of tobacco-addicted psychiatric inpatients – as hospitals become increasingly intolerant of smoking [9]. We set out to determine whether, as we suspected, psychiatric inpatients smoke more after admission than immediately beforehand and, if so, to explore their reasons.

The study was undertaken at a psychiatric hospital that serves a population of about 270 000 in northern England. Ethical approval was obtained from the North Yorkshire Research Alliance. Data were collected over three months in 2004–05. All in-patients aged 18 to 65 on the admission unit were invited to participate. Those who gave written consent completed a self-report questionnaire about their smoking since the week before admission. The questionnaire (available on request) was developed by the researchers as we were unable to identify any pre-existing tools that incorporated the data we wanted to collect. Those patients who reported a change since admission underwent a semi-structured interview to establish reasons; we stopped interviewing when emerging themes from answers reached saturation. Initially, open questions were used allowing free expression and capturing responses that we might not have expected. Participants then rated how much they agreed with a list of suggested reasons why they might be smoking a different amount (developed using reasons cited in published research, references available from authors upon request).

Expecting patients to smoke tobacco in various forms, we developed a cigarette-equivalent table using a tobacco price survey [10]; we determined that 1 small cigar, 2 roll-up cigarettes, and 3 grams of pipe tobacco were each equivalent to 1 manufactured cigarette. We categorised participants into light, moderate or heavy smokers using the Office for National Statistics' system [11]. We analysed quantitative data using SPSS version 12. We organised qualitative data into categories, then refined the categories and their properties.

During data collection there were 62 inpatients in the Unit; 7 were too ill to provide informed consent and 12 refused to participate. The 43 participants were aged 19–63 (median 38) years, 18 of them male (42%). In the week before admission, 24/43 (56%) reported smoking – rising to 30/43 (70%) after hospitalisation (McNemar chi squared, P = 0.03). The median number of cigarette equivalents each person smoked per day increased from 5 in the week before admission (interquartile range, IQR, 0–20) to 13 (IQR 0–20) in hospital (Wilcoxon Signed Ranks Test z = -2.387, P = 0.02); the increase was greater in males (increase from 6 to 15) than in females (from 6 to 12). We found a sharp rise in the number of people smoking moderately or heavily (10 or more cigarettes a day): 18 before admission, 27 afterwards (McNemar chi squared, P = 0.01).

Twenty-three people, 10 males and 13 females, completed the second stage of data-collection: 5 smoked less after admission and 18 smoked more. When the participants who smoked more were asked why, six overlapping categories emerged: boredom; stress; to socialise; habituation to smoking more; a substitute for unavailable alcohol; and a pleasant ambience to the smoke room.

We reduced these responses to three core categories (see Table 1 for quotes): boredom – most participants indicated that smoking was a way to pass the time; socialising – participants described going into the smoke room to be in the company of others and to make new friends, a more compelling reason to smoke than any direct effect of the cigarette; and stress reduction – a substantial majority asserted that stress, anxiety or the need to relax were reasons for smoking more after admission.

**Table 1:** Reasons for changes in smoking habits after admission – quotes from inpatients

A: Reasons for smoking more
**Boredom:**
"Boredom, especially between 12.15 and 5 o'clock when there's nothing else to do in the hospital; then after tea until visitors come it's mainly boredom" (m, 59); "Smoking gives you something to do" (f,32)
**Socialising**:
"I meet more people in the smoke room... I don't inhale so there's only a slight effect; it's more to do with socialising" (m, 38); "You go in the smoke room to talk to somebody and you end up putting a ciggy in your mouth" (f, 38)
**Stress reduction**
"My stress levels are up and I smoke to reduce them" (f, 22)

**B: Reasons for smoking less**

**Limited, unpleasant smoking areas**
"You can't just sit with everyone else together, you have to go to a separate room to smoke, you can't sit and watch TV whilst smoking" (f, 49); "It's not as convenient to smoke in here... sometimes it's uneasy in the smoke room, gets a bit claustrophobic in there" (f, 34)
**Improved mental state**
"I was very, very anxious before I came into hospital so I was smoking a lot more trying to calm me down" (m, 57)

In brackets are m/f for male/female, and the patient's age in years.

We identified five overlapping explanations for why a minority of participants smoked less after admission: an unpleasant smoke room; fewer places to smoke than they were used to; feeling less motivated to smoke; cutting down to discourage others from smoking; and improvement in mental state since admission.

We reduced these responses into two core categories (see Table 1 for quotes): unpleasant smoking areas – some respondents disliked having to sit away from the main lounge areas; and improved mental state – a few patients found that they smoked less because admission and treatment had resulted in greater feelings of self-control, less anxiety, and improved self-esteem.

In answer to our specific questions, participants did not think that medication side-effects were a reason for their change in smoking habits. There were only a few reports of a hospital 'economy' in which cigarettes were a form of currency. About a third of patients, regardless of whether they smoked more or less, felt pressurised into changing their smoking habit after admission. Few patients thought that their finances had significantly affected their smoking habits.

Even before hospitalisation, 56% of our patients were smoking, with 42% of the sample smoking moderately to heavily, well in excess of the British averages (30% smoking and 21% smoking moderately to heavily [11]). Our sample was small but it was reasonably representative, missing a minority of patients due to absence, illness, or refusal. The study was partly retrospective and thereby prone to recall bias, perhaps by socially acceptable responses. We could not find any tools for data collection so we developed and used non-validated instruments. Using self-report questionnaires may have lead to report biases. Despite limitations, it is plain that admission to the psychiatric unit was associated with a significant increase in the amount smoked.

Participants who smoked more after admission put it down to boredom and stress, with the smoke room offering a form of socialising. In contrast, the few people who were smoking less blamed the change on small, unpleasant smoke rooms and on the people who converged there. Some said that they were smoking less because their mental state had improved.

Psychiatric patients, partly because of medication side-effects, are prone to medical conditions that are worsened by smoking, such as metabolic syndrome [12]. Smoking has also been shown to increase tolerance of psychotropic drugs such as the neuroleptics. Consequently we want patients to smoke less rather than more after admission. The hospital smoke rooms are bare places with few chairs, designed to discourage patients from lingering. This may have discouraged a few smokers but a majority of our sample nonetheless saw the smoke room as a sociable place that they could escape to, diminishing the stresses and boredom of being in hospital.

If smoking is to lessen rather than intensify in mental health units, there is much to be done. Boredom and lack of therapeutic activity are widely recognised as the bane of inpatient wards; here is further evidence of the detrimental effect of low staffing and insufficiently active management of the ward milieu. Our study participants wanted to socialise, speak freely with other inpatients, and achieve some stress reduction; the challenge is to provide a smoke-free setting where these activities can flourish.

Lastly, we know that healthcare services are poor at pointing people towards smoking cessation interventions, despite effective services being available [13]. The gap between the health of the population as a whole and that of people with mental illness will widen if the mental health services fail to seize their educational opportunities.

## Competing interests

SK and DO are both non-smokers.

## Authors' contributions

SK devised the project with help from DO, as a research project for the MMedSc in Clinical Psychiatry, the University of Leeds. SK led the collection of data. Both authors had access to all data, analysed the data, read and approved the final manuscript; they take joint responsibility for its integrity and accuracy.
